# Development of a deep learning model to distinguish the cause of optic disc atrophy using retinal fundus photography

**DOI:** 10.1038/s41598-024-55054-0

**Published:** 2024-03-01

**Authors:** Dong Kyu Lee, Young Jo Choi, Seung Jae Lee, Hyun Goo Kang, Yu Rang Park

**Affiliations:** 1 Department of Ophthalmology, Institute of Vision Research, Severance Eye Hospital, Yonsei University College of Medicine, Yonsei-ro 50-1, Seodaemun-gu, Seoul, 03722 Republic of Korea; 2 Department of Biomedical Systems Informatics, Yonsei University College of Medicine, Yonsei-ro 50-1, Seodaemun-gu, Seoul, 03722 Republic of Korea

**Keywords:** Deep learning, Fundus photography, Leber hereditary optic neuropathy, Optic neuritis, Optic neuropathy, Translational research, Data processing, Machine learning

## Abstract

The differential diagnosis for optic atrophy can be challenging and requires expensive, time-consuming ancillary testing to determine the cause. While Leber's hereditary optic neuropathy (LHON) and optic neuritis (ON) are both clinically significant causes for optic atrophy, both relatively rare in the general population, contributing to limitations in obtaining large imaging datasets. This study therefore aims to develop a deep learning (DL) model based on small datasets that could distinguish the cause of optic disc atrophy using only fundus photography. We retrospectively reviewed fundus photographs of 120 normal eyes, 30 eyes (15 patients) with genetically-confirmed LHON, and 30 eyes (26 patients) with ON. Images were split into a training dataset and a test dataset and used for model training with ResNet-18. To visualize the critical regions in retinal photographs that are highly associated with disease prediction, Gradient-Weighted Class Activation Map (Grad-CAM) was used to generate image-level attention heat maps and to enhance the interpretability of the DL system. In the 3-class classification of normal, LHON, and ON, the area under the receiver operating characteristic curve (AUROC) was 1.0 for normal, 0.988 for LHON, and 0.990 for ON, clearly differentiating each class from the others with an overall total accuracy of 0.93. Specifically, when distinguishing between normal and disease cases, the precision, recall, and F1 scores were perfect at 1.0. Furthermore, in the differentiation of LHON from other conditions, ON from others, and between LHON and ON, we consistently observed precision, recall, and F1 scores of 0.8. The model performance was maintained until only 10% of the pixel values of the image, identified as important by Grad-CAM, were preserved and the rest were masked, followed by retraining and evaluation.

## Introduction

Leber’s hereditary optic neuropathy (LHON) is a hereditary mitochondrial disease that is characterized by acute or subacute visual loss usually involving both eyes, and typically occurs between the ages of 15–35 years^1^. LHON is a relatively rare disease, with the prevalence of vision failure caused by LHON reported to be approximately 3.22 per 100,000 (95% CI 2.47–3.97 per 100,000) in the UK^2^. In the acute phase of the disease, the optic nerves are hyperemic with swelling of the peripapillary nerve fiber layers. Over time, the disc edema usually improves, although with subtle signs of optic disc atrophy^3^. However, these characteristics are not specific for LHON; it may be difficult to distinguish from normal optic discs in the early stages, and may be confused in the later stages with various diseases that can cause optic neuropathy, including diseases such as demyelinating optic neuritis (ON), toxic optic neuropathy, and dominant optic neuropathy^4^. Thus, delayed referrals leading to late initial visits to relevant specialists may present challenges in the differential diagnosis. While certain disc features can help determine the cause, such as sector disc pallor in older individuals suggesting non-arteritic ischemic optic neuropathy, severe optic atrophy with gliosis in elderly patients suggesting giant cell arteritis, these signs are not as definitive and distinct in the vast majority of cases^5^. However, determining the causative factor is crucially important as optic atrophy may indicate a wide variety of inflammatory, infectious, toxic, neoplastic, or genetic causes. In fact, there have been case reports of misdiagnosed patients in the literature showing the need for better methods to distinguish the causative factor for optic disc atrophy^6,7^. The differential will thus require a sometimes exhaustive list of ancillary, time-consuming, and expensive testing.

Over the past years, deep learning (DL) models have been applied to a variety of medical problems, with astonishing success in certain areas pertaining to medical imaging classification^8,9^. In the ophthalmic field, convolutional neural networks (CNNs) can differentiate not only typical macular diseases such as diabetic retinopathy or age-related macular degeneration^10,11^ but can also accurately predict the age, sex, and cardiovascular disease risk^12,13^. In addition, neurologic diseases can be differentiated by optic disc changes: we have recently shown that DL-based screening models appear able to differentiate autism spectrum disorder from neurotypical children based on retinal fundus photography^14^. While these aforementioned studies require large datasets for adequate training of DL models, recent studies have shown that the ResNet-18 model, an enhanced DL algorithm based on a CNN, can be trained even with small sample sizes, such as chorioretinal atrophy or inherited retinal disorders^15,16^. Reliable training with smaller datasets are important for rare disease entities such as for LHON and ON, since both relatively rare in the general population, contributing to limitations in obtaining large imaging datasets.

Thus, in this study, we aimed to develop a DL model that can distinguish the cause of optic nerve atrophy between LHON and ON by using only retinal fundus photography.

## Methods

The data collected in this study consisted of patients diagnosed at Severance Eye Hospital affiliated with the Yonsei University College of Medicine, which is a tertiary, referral-based, high-volume hospital. This study was approved by the Severance Hospital Institutional Review Board (IRB Approval No. 4-2023-0221) and adhered to the tenets of the Declaration of Helsinki. The requirement for informed consent was waived owing to the retrospective nature of the study by the Severance Hospital IRB.

To include only those patients with confirmed causes of optic disc atrophy, those diagnosed with either LHON or ON between January 2010 and December 2022 were included in the analysis. Patients diagnosed with LHON both clinically through multimodal imaging and with confirmed mitochondrial mutations by next-generation sequencing (NGS) were included; retinal fundus photography images at 6 months after initial presentation (after regression of disc hyperemia) were included in the dataset. Patients diagnosed with ON through multimodal imaging (including orbital magnetic resonance imaging and delayed P100 on visually evoked potential testing) were included, and retinal fundus photography images at 6 months after initial presentation were used in the dataset. Suspected LHON patients without genetically confirmed mutations and ON-suspected patients with missing multimodal imaging were excluded. Therefore, the dataset comprised simulated delayed presentations of patients with optic atrophy with confirmed causes. For comparison with the normal population, fundus images were collected from patients without any relevant retinal or systemic diseases through a general health checkup center.

### Preprocessing of retinal photography

**Figure 1:**
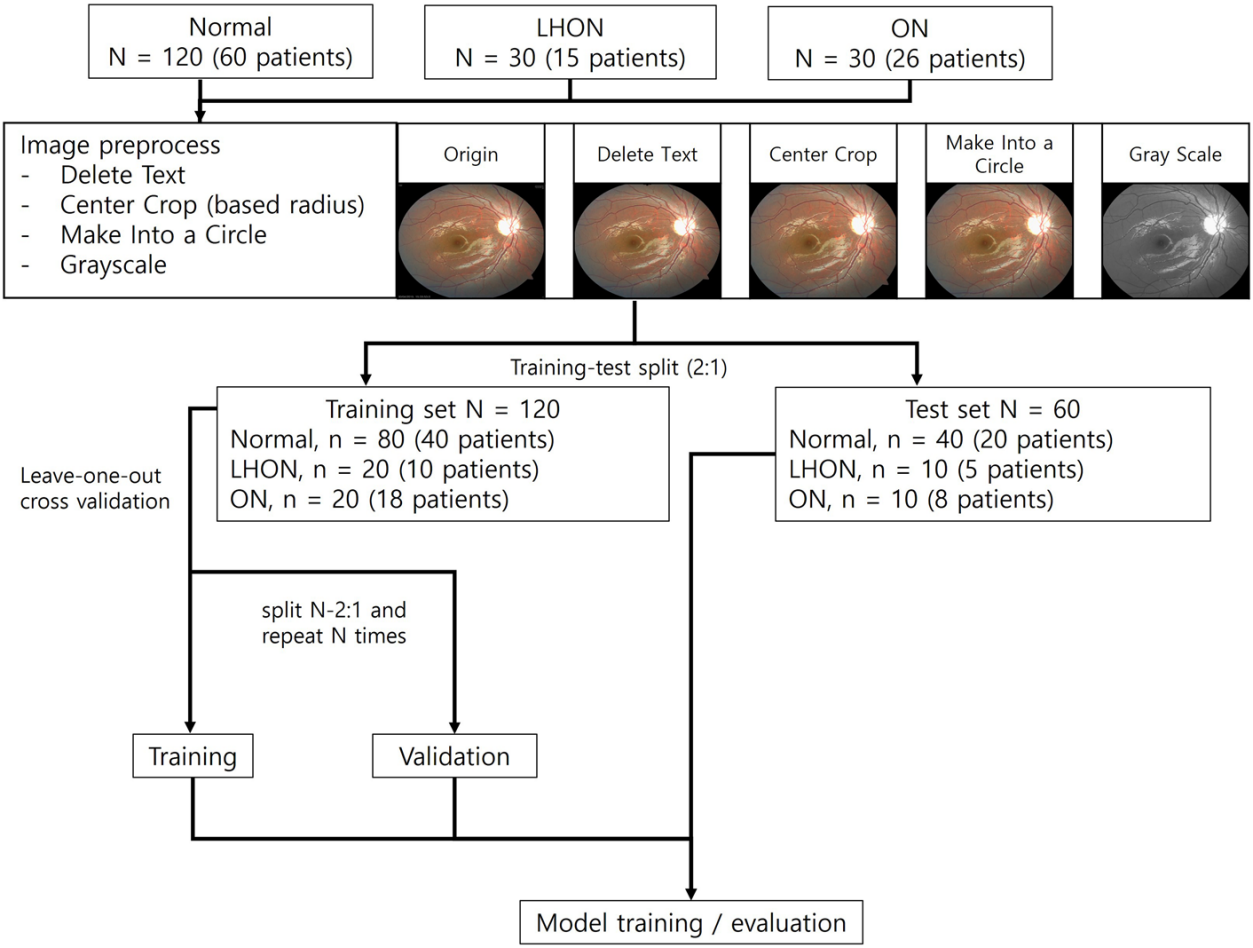
Overview of the proposed study flow.

Each retinal image underwent the following preprocessing steps for use as an input for the model (Fig. 1). First, the text at the corners of the image was removed to prevent the model from examining unnecessary areas. In this process, OCR was used and the area of the detected text was filled with RGB^2^. Subsequently, the center of the retina was cropped for consistency, as some images contained all areas of the retina and some contained only the cut areas. The radius was determined and some ratios on the left and right were cut off. In addition, each retinal image includes a tip, but the locations of the tip of the entire image are different, and some were cut during the cropping process; therefore, the tip part was removed to form a circular shape. When it was placed in the model, it was changed to grayscale and proceeded. Before inputting the preprocessed images into the model, we applied a resizing method to create images of 224 × 224 pixels, which are already used in the pre-trained model and will therefore facilitate parameter optimization.

### DL model development and evaluation

The preprocessed image was divided into training and test sets at a ratio of 2:1 while maintaining the target class ratio. To avoid data leakage during the training-test split process, paired retinal images of the same person were designed to belong to the same set. The overall process of the model training and testing was carried out in three target classes: normal, LHON, and ON. Because the amount of training data was small, a separate validation set was not configured. Instead, leave-one-out cross validation was performed to evaluate the model that learned with N − 2 or N − 1 images (except for the paired retinal images of the same person) out of the total training set of N images with the remaining one image, and the process was repeated N times. After the leave-one-out cross validation was completed, the entire training dataset was used to model the retraining with the obtained hyperparameters from leave-one-out cross validation, and the scores of the test set were obtained.

The model used was ResNet-18 from PyTorch^17,18^. The model was pretrained with Imagenet-1000. The utilization of a pre-trained model, which is already trained to recognize a wide range of patterns from large datasets, aims to avoid overfitting to our small training dataset along with improving generalization performance. All layers, including the input and output layers which were newly defined according to the number of channels and classes, respectively, were fine-tuned using our retinal photographs. Details regarding hyperparameter optimization and risk of over-fitting with ResNet-50 can be found in the Supplements. Briefly, we used the cross-entropy loss function and the other hyperparameters of fine-tuning: the learning rate was 1e−5, batch size was 16, number of epochs was 30, and the optimizer was Adam with default parameters of β1 = 0.9 and β2 = 0.999. In this study, we measured the area under the receiver operating characteristic (AUROC). The receiver operating characteristic (ROC) curve shows a trade-off between recall and 1-specificity. The area under this curve summarizes the diagnostic ability of the model for each threshold. The AUROC is a score for binary classification and cannot be calculated directly from multiclass classification. Therefore, we calculated each AUROC for the cases obtained by treating each class as positive (one-vs.-rest). To reduce false negatives, various metrics such as recall (or sensitivity), which is the correctly predicted ratio among positive samples; precision, which is the ratio of true positives among samples predicted to be positive; and F1 score, which is a harmonic mean, were used. These metrics were measured using a one-versus-rest method, such as AUROC, but the one-versus-one method measured only with data that are two classes in the true label was also used.

### Visualization method

To visualize the critical regions in retinal photographs that are highly associated with disease prediction, Gradient-Weighted Class Activation Map (Grad-CAM) was used to generate image-level attention heat maps and to enhance the interpretability of the DL system^19^. Grad-CAM calculates feature weights to generate attention heat maps that highlight the regions that play an important role in the final prediction. By visualizing image-level attention heat maps, we can observe and determine the contribution of potential fundus features to the final disorder prediction.

We used progressive erasure plus progressive restoration (PEPPR) to numerically investigate whether the model focused on key areas for classification^20^. Because comparing and validating each image with the attention heat map obtained by Grad-CAM is time-consuming and requires domain experts, we aggregated these image-specific descriptions to determine if the model had a global explanation for the entire image. Based on the attention obtained by Grad-CAM, we gradually removed the less important parts of each image and examined the performance of the model. The determination of important and less important areas was based on the value of the element in the attention heatmap, which was divided into 10 sections in ascending order of importance. A model with the same hyperparameters as the original model was re-taught using a new training set obtained by masking all pixels whose values were less than the elements of a given section. Subsequently, to verify that the identified attention region was still effective in the disease classification task, the classification performance (AUROC) was measured with a similarly masked test set. This method of visualization will enable us to determine and validate the region of highest interest that the model is using for classification, thus enabling clinicians to understand the model’s decision-making process.

### Statistical analyses

Descriptive statistics were used to characterize the study populations. As normally distributed data could not be assumed because of the small dataset, nonparametric tests including the Mann–Whitney U test, chi-square/Fisher’s exact test, and linear-by-linear association were performed. Descriptive and statistical analyses were performed using SPSS version 25.0 (IBM Corp, Armonk, NY, USA) and RStudio version 1.4.1106 (R version 4.0.4; Boston, MA, USA). *P* < 0.05 was considered statistically significant.

### Ethics statement

This study was approved by the Severance Hospital Institutional Review Board (Approval Number: 4-2023-0221) and adhered to the tenets of the Declaration of Helsinki. The requirement for informed consent was waived owing to the retrospective nature of the study by the Severance Hospital Institutional Review Board.

## Results

### Basic characteristics

The dataset used in this study consisted of 30 eyes with LHON from 15 patients, 30 eyes with ON from 26 patients, and 120 normal eyes from 60 patients. All patients were ethnically Korean. The baseline characteristics of the LHON and ON groups, including gender distribution, are summarized in Table 1. All patients with LHON had bilateral involvement, whereas there were only four cases of bilateral involvement in the ON group. The mean age at the time of diagnosis in the LHON and ON groups was 28.3 years and 47.1 years, respectively. The most common mitochondrial mutation detected by NGS in the LHON group was the 11,778 variant (8 patients), followed by the 14,484 variant (6 patients), and 3460 variant (1 patient). There were no significant differences between the two groups in terms of the visual field indices, retinal nerve fiber layer (RNFL) thickness, and ganglion cell layer thickness on optical coherence tomography (OCT) (all *P* > 0.05; Mann–Whitney U test).

**Table 1:** Baseline characteristics.

	Leber’s hereditary optic neuropathy	Optic neuritis	*P*-value^a^
No. of patients (eyes)	15 (30)	26 (30)	–
Sex (male/female)	10/5	12/14	–
Diagnosis age	28.3 ± 18.51	47.1 ± 19.7	–
Laterality (Right/Left)	15/15	10/20	–
Visual field 30–2 mean deviation (dB)	− 9.38 ± 9.24	− 7.02 ± 10.79	0.536
Retinal nerve fiber layer thickness (μm)^b^	76.87 ± 13.01	80.91 ± 18.69	0.725
Ganglion cell Layer thickness (μm)^c^	69.56 ± 14.36	75.95 ± 15.98	0.675

^a^Mann–Whitney U test.

^b^A macular scan of each participant’s eye was obtained using the macular cube 200 × 200 scan protocols. Ganglion cell-inner plexiform layer thickness (GCIPL) values were obtained using the ganglion cell analysis algorithm from an elliptical annulus localized on the fovea center; the average GCIPL thickness were measured.

^C^The average retinal nerve fiber layer thickness measurements were also assessed with the 6 mm × 6 mm data cube captured by the Optic Disc Cube 200 × 200 scan (Cirrus™; Carl Zeiss Meditec, Inc., Dublin, CA, USA).

### DL model for screening LHON and ON

**Figure 2:**
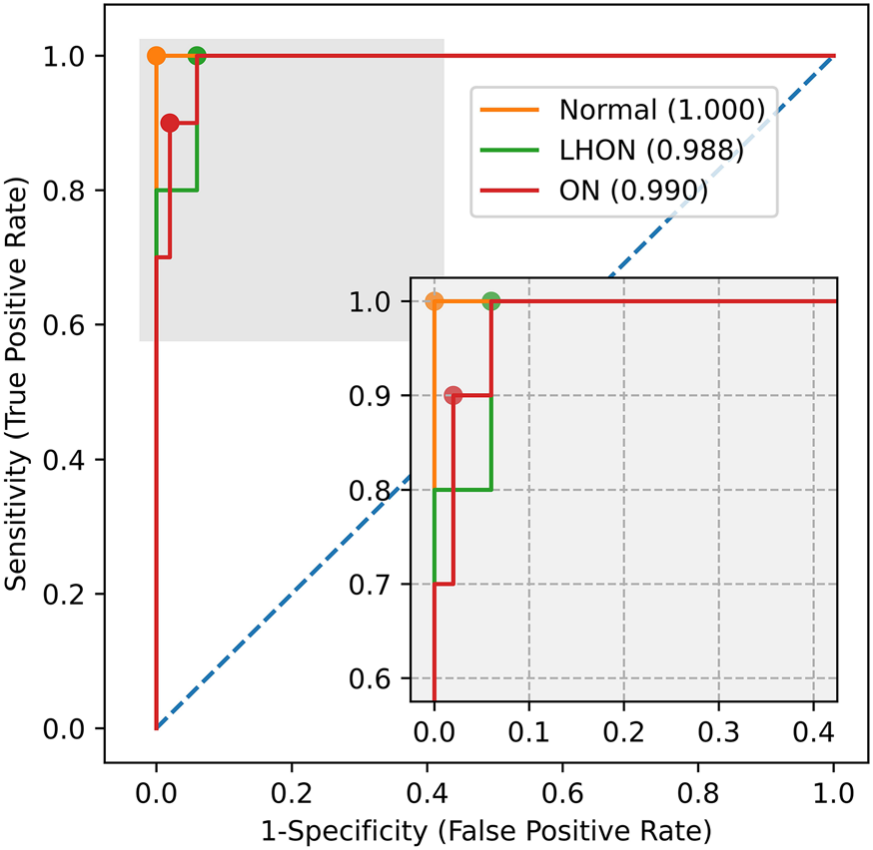
Receiver operating characteristic curves. The yellow curve represents how the model distinguishes normal from other classes, the green curve represents how the model distinguishes LHON from other classes, the red curve represents how the model distinguishes ON from other classes. The AUROC is represented in each legend. LHON, Leber’s hereditary optic neuropathy; ON, optic neuritis; AUROC, area under the receiver operating characteristic curve.

Figure 2 illustrates the ROC curve superimposed on the performance of the model for the normal, LHON, and ON classifications. Classification of normal from disease was the best with an AUROC of 1.0, a sensitivity of 1.0, and a specificity of 1.0, followed by classification of ON from other classes with an AUROC of 0.990, a sensitivity of 0.9, and a specificity of 0.98, and classification of LHON from other classes with an AUROC of 0.988, a sensitivity of 1.0, and a specificity of 0.94.

**Figure 3:**
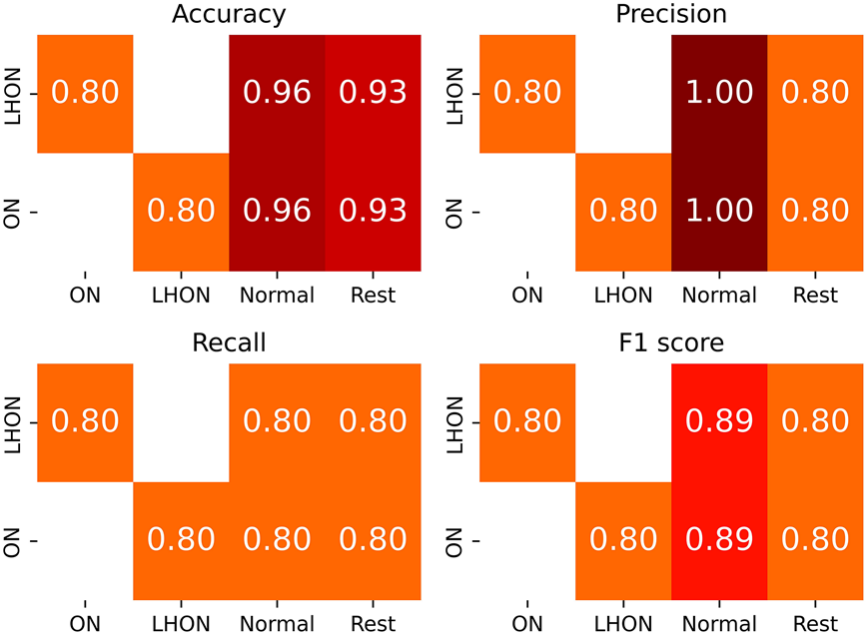
Heatmap of accuracy, precision, recall, and F1 score of model between classes. LHON, Leber’s hereditary optic neuropathy; ON, optic neuritis. A comparison between LHON and “Rest” is a comparison between LHON and both ON and normal.

Figure 3 is a pairwise heatmap representing the accuracy, precision, recall, and F1 score, and includes metric values for pairs of all classes. The performance of classifying each disease from different classes was calculated as an accuracy of 0.93 and precision, recall, and F1 score of 0.8 for both LHON and ON. For distinguishing between LHON and ON, the accuracy, precision, recall, and F1 score were calculated to be 0.8. This shows that even for the purpose of classifying between LHON and ON diseases, the model can achieve a high performance.

**Figure 4:**
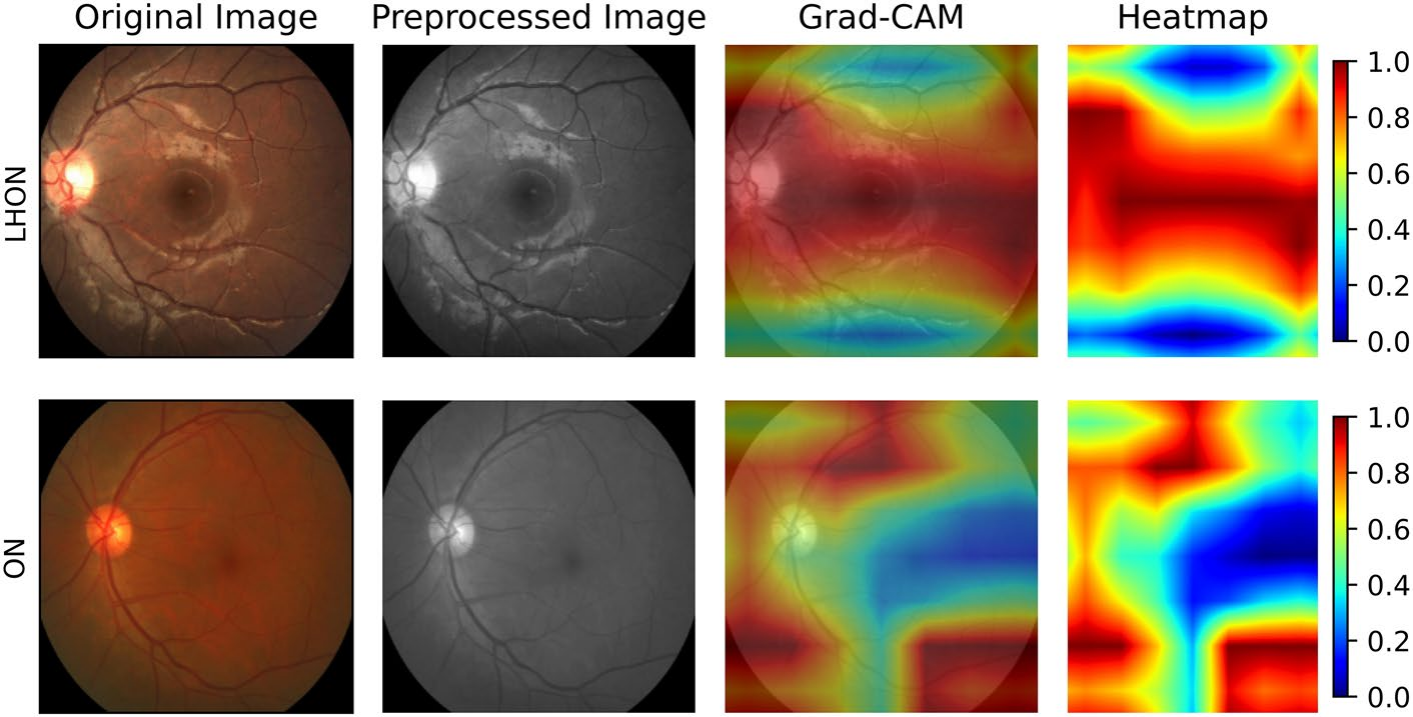
Representative retinal fundus photographs. In each row, the first image shows the images after cropping, the second image shows the input of the model, the third image shows which areas the model paid attention to, and the fourth image is the heatmap of Grad-CAM and shows the intensity of attention. Grad-CAM, Gradient-Weighted Class Activation Map; LHON, Leber’s hereditary optic neuropathy; ON, optic neuritis.

To distinguish between LHON and ON, the visualization of which anatomical part that the model viewed and predicted the class is shown in Fig. 4. Grad-CAM uses the gradient of all the output classes flowing from the model to the final convolution layer to obtain a map for the critical regions of the image for prediction. The figure represents the importance of each pixel obtained through Grad-CAM as a heat map and is superimposed on the original image to determine the location where the model is focused. The model shows disease-specific attention patterns. For LHON, the model focuses primarily on the optical disc and macular region, with a particular emphasis on macular grass bundles. However, when ON is displayed in the fundus image, the model's attention shifts around the upper and lower arcades.

### Validating global attention maps

**Figure 5:**
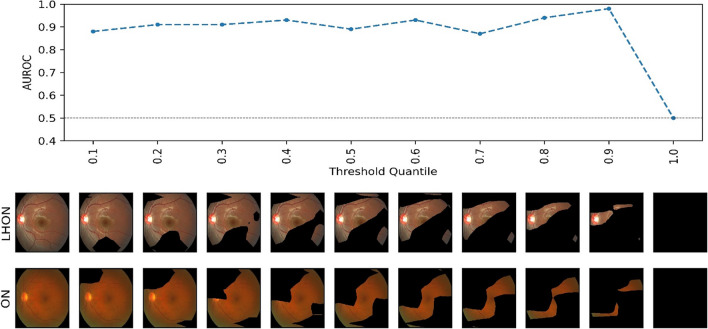
Progressive erasure for each model, and AUC scores from the test set under the blurred images whose attention heatmap values are under the specific quantile. AUROC, area under the receiver operating characteristic curve; LHON, Leber’s hereditary optic neuropathy; ON, optic neuritis.

We also utilized PEPPR by using attention heatmaps from Grad-CAM and masking the pixels with values below a certain threshold. A model with hyperparameters identical to those of the original model was re-taught and evaluated using the images obtained by masking. We performed this process progressively for masking thresholds ranging from < 10 to < 100%. Figure 5 illustrates the AUROC performance of the model for different levels of masking. We observed that even when only 10% of the important regions were retained (with 90% masking), the AUROC performance remained unaffected.

## Discussion

Distinguishing the cause of optic nerve atrophy is a challenging task, even for relevant specialists, and may require extensive ancillary testing, with the potential to cause undue financial burden. Even if LHON is suspected, tests such as OCT, visual fields, electroretinograms, and visually evoked potentials are not only necessary, but additional blood work including NGS may be necessary for confirmation^21–23^. Additionally, ancillary testing including brain magnetic resonance imaging (MRI), spinal MRI, cerebrospinal fluid studies, and AQP4 and MOG antibody tests may be performed to rule-out multiple sclerosis for neuromyelitis optica in cases with ON^24,25^. All of these tests are time-consuming, expensive, and cannot be performed in the clinic, which requires referral to a specialist, causing another delay in diagnosis. Our study demonstrated the possibility of automated DL classification between LHON and ON using only non-invasive, relatively inexpensive, and repeatable fundus photography.

Our study showed a high precision and recall score, with an accuracy of 0.9. LHON is mainly caused by mitochondrial mutations at G11778A, G3460A, and T14484C and results in a decrease in ganglion cells and RNFL thickness^1^. In early stages of the disease, telangiectatic microangiopathy and disc edema are observed in the early stages of the disease. In OCT, RNFL thickness was increased in the 360° average measurement, and the most significant increase in RNFL thickness was observed in the superior quadrant. In contrast, no significant changes were detected in the temporal quadrant, which is in line with the early loss of fibers in the papillomacular bundle^26,27^. In the atrophic stages of LHON, the peripapillary RNFL was thinned in all quadrants, and the temporal quadrant was the first and the most severely affected^3^. Accordingly, by using Grad-CAM, we were able to visualize areas that the model relied on to predict the results. For LHON, our model appears to classify mitochondrial disease by focusing on changes in the area around the optic disc head and the papillomacular bundle, showing a link to the pathomechanism of injury in LHON patients.

In contrast, the highlighted areas of ON eyes were mainly the optic disc head and vascular arcades. In fact, several studies have previously shown that the peripapillary RNFL and papillomacular bundles appear to be thinned in the atrophic stages of ON^18,28^. Additionally, with OCT angiography, Yu et al. and Huang et al. showed that the peripapillary vessel density of ON patients was significantly reduced, implying that ON progression may lead to changes in the retinal vessels^18,29^. This is in line with the fact that Grad-CAM significantly identified the area around the vascular arcades in our study. Finally, our results from Table 1 may further support these findings, as macular changes and visual field indices did not appear to be significantly different between groups; thus, alterations specific to the disc and vascular arcades may be more important areas of focus for differentiation between LHON and ON. Data-driven methods used to identify informative regions can also be applied to validate prediction models for rare ocular diseases. This data-derived method has been applied in several previous ocular disease predictions^20,30^, but it has not been presented in rare diseases such as our study. This study confirmed that the performance of the model did not decrease even when 90% of the unimportant parts of the image were gradually erased. This indicates that the attention map correctly reflects the regions that are most important to the model. However, it is surprising that having only the most important 10% of the retinal images is sufficient to obtain a performance comparable to that of the full images available. In summary, we found that the global attention map faithfully reflects how the model works. Surprisingly, 10% of the images containing the posterior pole were sufficient to achieve high performance.

While the effects of camera- and population-specific traits affecting model performance, which may reduce model generalizability, should always be considered, two published studies comparing the performances in fundus photo-specific DL models showed that these effects may be limited in scope. Jimmy et al^31^. demonstrated that a US-data trained DL model had AUROC change from 0.99 to 0.96 when validated using a Nepal-obtained dataset, indicating that a high-quality dataset appears to be able to construct a DL model with performance that remains consistent despite input of data from other countries and therefore different fundus cameras. Additionally, Ryo et al.^32^ showed that, at least in an ophthalmology clinic using fundus photo-specialized cameras, camera-specific traits may have only a limited or no effect on DL model performance.

Thus, while the differential diagnosis for optic nerve atrophy can be challenging, the results of our study show that DL-based models may assist clinicians in reducing the need for unnecessary ancillary testing. In fact, several studies have shown that retinal fundus photography-based models may be particularly adept at finding subtle but clinically important alterations to the optic disc that may signal a variety of optic nerve and neurologic diseases. While the majority have mainly dealt with the glaucomatous optic neuropathy^32–37^, there has been one study showing good performance in DL models differentiating between optic neuropathies and pseudo-papilledema^38^, and a recent study from our group showing the ability to screen for autism spectrum disorder in children based mainly on optic disc and peripapillary retinal changes^14^. Our study furthers this effort by proving that even using smaller datasets for rare diseases can be utilized to train and differentiate between subtle disc and retina changes that may be otherwise undetectable by the human eye. As retinal fundus photography is a rapid, non-invasive, well-tolerated, repeatable, relatively inexpensive imaging technique that is widely available in general ophthalmology clinics, our findings show that a single, relatively affordable fundus photo can be used to eliminate the need for expensive ancillary testing.

Our study has several limitations owing to the limited size of the dataset; additionally, the severity of the diseases was not reflected in the model training. However, given that NGS-confirmed LHON and ON are relatively rare and that the DL model used in this study appears to show good performance even in smaller datasets in the published literature, we believe that our results are significant and hypothesis-generating for a future multicenter study. Finally, our particular strategy for validation may cause concern for model generalizability and risk of overfitting. However, the leave-one-out cross validation allowed us to use all the data as a validation set once, while keeping the number of the training dataset approximately the same size; this therefore allowed us to find the hyperparameter with the best performance for generalization even while limited to a small dataset. Additionally, the final performance was based on a test set that was never used in the cross-validation, limiting the risk of overfitting.

In conclusion, we confirmed that DL is able to distinguish subtle optic disc atrophy caused by NGS-confirmed LHON and ON with relatively good performance. Further development of this model can provide a highly cost-effective and reliable alternative to the extensive and time-consuming ancillary testing currently performed in real-world clinics, even in cases with delayed referrals.

### Supplementary Information

Supplementary Information.

## Data Availability

The datasets used and analysed during the current study available from the corresponding author on reasonable request.

## Abbreviations

AUROC: Area under the receiver operating characteristic curve
CNN: Convolutional neural network
DL: Deep learning
LHON: Leber’s hereditary optic neuropathy
MRI: Magnetic resonance imaging
NGS: Next-generation sequencing
OCT: Optical coherence tomography
ON: Optic neuritis
PEPPR: Progressive erasure plus progressive restoration
RNFL: Retinal nerve fiber layer
ROC: Receiver operating characteristic
