# Supplementary material for: Development of a deep learning model to distinguish the cause of optic disc atrophy using retinal fundus photography
Source: Sci Rep. 2024 Mar 1;14:5079. doi: 10.1038/s41598-024-55054-0 (PMC10907364; doi:10.1038/s41598-024-55054-0)
Supplement: Supplementary file 1 — Supplementary Information. [file 41598_2024_55054_MOESM1_ESM.pdf]

SUPPLEMENTS

Hyperparameter optimization

Multiple combinations of hyperparameters were exhaustively evaluated using leave-one-out cross-validation. The evaluated combinations included the decision of using a grayscale, a combination of green and blue colors from the RGB scale (with red excluded), or the full RGB scale for the color scale. Additionally, various settings for learning rate, batch size, and epoch number were explored, along with different hyperparameters for the Adam optimizer. After conducting extensive tests, we identified the combination that yielded the best performance. We used a gray scale for the color scale, a learning rate of 10e-5, batch size of 16, 30 epochs, and Adam optimizer settings with beta1 and beta2 values of 0.9 and 0.999, respectively.

Performances from ResNet-50

We utilized a shallow layer ResNet-18 model to better suit our limited dataset. When using a deeper layer ResNet-50 model, we achieved the following results: accuracy of 0.8667, precision of 0.7361, recall of 0.7333, and an F1 score of 0.731. To differentiate normal from other cases, we achieved an accuracy of 0.8667, precision of 1.0, recall of 1.0, F1 score of 1.0, and an AUROC of 1.0. For LHON from other cases, we achieved an accuracy of 0.8667, precision of 0.625, recall of 0.5, F1 score of 0.556, and an AUROC of 0.966. For ON from other cases, we achieved an accuracy of 0.8667, precision of 0.5833, recall of 0.7, F1 score of 0.6364, and an AUROC of 0.97.
